# Ready for Safe Cancer Treatment (RESET): Protocol for a Large-Scale Randomized Controlled Trial of an Integrated Perioperative Care and Safe-Discharge Pathway in Oncological and Older Surgical Patients

**DOI:** 10.3390/mps9050134

**Published:** 2026-09-18

**Authors:** Natalia Jędruchniewicz, Hanna Klimza, Marek Zawadzki, Mariola Dwornikowska-Dąbrowska, Bernard Zając, Tomasz Klimek, Mariusz Kiszka, Mariusz Chabowski, Dorota Kamińska, Beata Jankowska-Polańska, Wojciech Witkiewicz, Małgorzata Wierzbicka

**Affiliations:** 1 Research and Development Centre, Regional Specialist Hospital in Wroclaw, 51-124 Wroclaw, Poland; natalia.jedruchniewicz@wssk.wroc.pl (N.J.); marek.zawadzki@wssk.wroc.pl (M.Z.); mariola.dwornikowska-dabrowska@wssk.wroc.pl (M.D.-D.); tomasz.klimek@wssk.wroc.pl (T.K.); wojciech.witkiewicz@wssk.wroc.pl (W.W.); 2 Department of Otolaryngology, Regional Specialist Hospital in Wrocław, Research and Development Centre, 51-124 Wrocław, Poland; wierzbicka.otolaryngology@gmail.com; 3 Department of Clinical Surgical Sciences, Faculty of Medicine, Wroclaw University of Science and Technology, 51-124 Wroclaw, Poland; mariusz.chabowski@pwr.edu.pl; 4 Department of Public Health, Faculty of Health Sciences, Medical University of Silesia, 51-124 Wroclaw, Poland; 5 Department of Anesthesiology and Intensive Care, Regional Specialist Hospital in Wroclaw, 51-124 Wroclaw, Poland; bernard.zajac@wssk.wroc.pl; 6 Innovation and Education Center, 4th Military Clinical Hospital, 51-124 Wroclaw, Poland; mkiszka@4wsk.pl (M.K.); bpolanska@4wsk.pl (B.J.-P.); 7 Department of Surgery, 4th Military Clinical Hospital, 51-124 Wroclaw, Poland; dkaminska@4wsk.pl; 8 Department of Non-Procedural Clinical Sciences, Faculty of Medicine, Wroclaw University of Science and Technology, 51-124 Wroclaw, Poland

**Keywords:** prehabilitation, prolonged hospitalization, rehospitalization, surgery, transitional care, Patient Outcomes

## Abstract

**Background:** Prolonged hospitalization, postoperative complications, and unplanned readmission remain major problems after major surgery. RESET evaluates a continuous perioperative pathway integrating individualized prehabilitation, coordinated inpatient care, enhanced-recovery principles, discharge planning, and transitional care in adults undergoing oncological surgery and patients aged 70 years or older undergoing non-oncological surgery. **Methods:** RESET is a prospective, two-center, two-arm randomized controlled study (NCT07727876). Up to 12,800 patients will be screened and approximately 8960 randomized 9:1 to RESET or standard care, including about 5740 oncological and 3220 non-oncological participants. The three primary outcomes are prolonged index hospitalization (≥14 days), unplanned readmission within 30 days after discharge, and postoperative complications within 30 days after surgery. Each outcome is evaluated separately in both clinical populations, yielding six primary hypothesis tests. Holm’s procedure will control the family-wise error rate at α = 0.01 across all six tests, with endpoint- and population-specific confirmatory conclusions. Secondary outcomes include length of stay, multidomain health status, satisfaction, adherence, organizational effectiveness, and direct healthcare costs. **Conclusions:** RESET will evaluate the clinical and implementation effects of an integrated perioperative pathway across two surgical populations.

## 1. Introduction

Prolonged hospitalization, unplanned readmission, and postoperative complications remain major clinical and organizational problems after major surgery, particularly in patients with cancer and in older adults with limited physiological reserve. Prehabilitation aims to improve surgical resilience through physical conditioning, nutritional optimization, and psychosocial support, but its clinical effects vary across populations and programs [1,2,3,4,5,6,7,8,9,10,11,12,13,14,15,16]. Frailty, sarcopenia, malnutrition, and impaired functional capacity further influence treatment tolerance and recovery, supporting multidomain assessment in both oncological and older surgical populations [17,18,19,20,21,22,23,24,25,26].

Variation in benefit likely reflects differences in baseline physiological reserve, multimorbidity, malnutrition and deconditioning, the short preoperative interval often available in oncology, and heterogeneity in intervention intensity, supervision, adherence, patient selection, and outcome definitions [1,7,9,11,12,15,16]. In addition, many existing approaches address only one phase of the perioperative pathway. Risk prediction, discharge planning, pharmacological review, transitional care, and resource-allocation models may reduce avoidable readmission or delay but are commonly implemented as separate rather than coordinated processes [27,28,29,30,31].

RESET was therefore designed as a continuous perioperative care pathway rather than as a stand-alone prehabilitation program. Individualized physical, nutritional, psychological, and pharmacological optimization begins approximately three weeks before surgery and continues through hospital admission, enhanced-recovery-based inpatient care, discharge planning, and 30-day transitional care. The pathway also incorporates specialist coordination, adherence monitoring, structured patient-flow tools, and post-discharge follow-up.

At the health-system level, coordination of cancer care in Poland is increasingly formalized through the National Oncology Network [32], but perioperative optimization still spans activities that are often delivered separately. RESET addresses this operational gap by linking prehabilitation, inpatient recovery, discharge planning, and post-discharge support within one protocolized but individualized pathway. To our knowledge, no widely adopted model in the participating health system combines these elements with structured patient-flow tools and 30-day transitional care as a single continuous perioperative pathway. The novelty of RESET therefore lies not in any one component, but in the prospective randomized evaluation of their coordinated delivery across the full perioperative continuum.

The study includes two predefined clinical populations: adults undergoing radical oncological surgery and patients aged ≥70 years undergoing elective non-oncological surgery. Both populations follow the same overall study framework but will be analyzed separately. The central clinical question is whether assignment to this integrated pathway improves clinically relevant and organizational outcomes compared with standard perioperative care.

The primary objective is to determine whether assignment to the integrated RESET pathway, compared with standard perioperative care, reduces the occurrence of: (1) prolonged index hospitalization, (2) unplanned hospital readmission within 30 days after discharge, and (3) postoperative complications. Each outcome will be evaluated separately within each of the two predefined clinical populations, yielding six primary hypothesis tests rather than a co-primary framework requiring simultaneous significance of all three outcomes. Confirmatory conclusions will be made only for the specific outcome-population hypotheses that satisfy the multiplicity-controlled decision rule specified in the Statistical Methods section of this paper. The secondary objectives are to:

- Evaluate the length of index hospital stay;
- Evaluate changes in multidomain overall health status, including patient-reported, laboratory, nutritional, anthropometric, psychological, and physical-function measures;
- Assess patient satisfaction and adherence to the individualized prehabilitation pathway;
- Evaluate the effectiveness of Stochastic Organizational Support Network Planning in facilitating specialist consultations;
- Compare direct healthcare costs associated with RESET and standard care;
- Assess implementation, continuity of care, and the potential scalability of the integrated pathway.

## 2. Materials and Methods

### 2.1. Study Design

This is a prospective, two-center, two-arm, parallel-group randomized controlled study evaluating the RESET perioperative care pathway in two predefined surgical populations. Participants who provide written informed consent and meet the eligibility criteria are randomized in a 9:1 ratio to the RESET intervention or standard-care control arm. Exploratory analyses may additionally use routinely collected clinical outcomes from a naturally formed group of eligible patients who do not participate, as prespecified in the approved protocol. The study was approved by the Bioethics Committee at the Lower Silesian Medical Chamber in Wroclaw (approval No. 33/BNBO/2025, 12 November 2025). Protocol version 1.2, dated 27 March 2026, was developed in accordance with the SPIRIT 2013 Statement (Additional File 1) [33]. The study is being conducted at the Regional Specialist Hospital in Wroclaw and the 4th Military Clinical Hospital with Polyclinic in Wroclaw.

**Figure 1:**
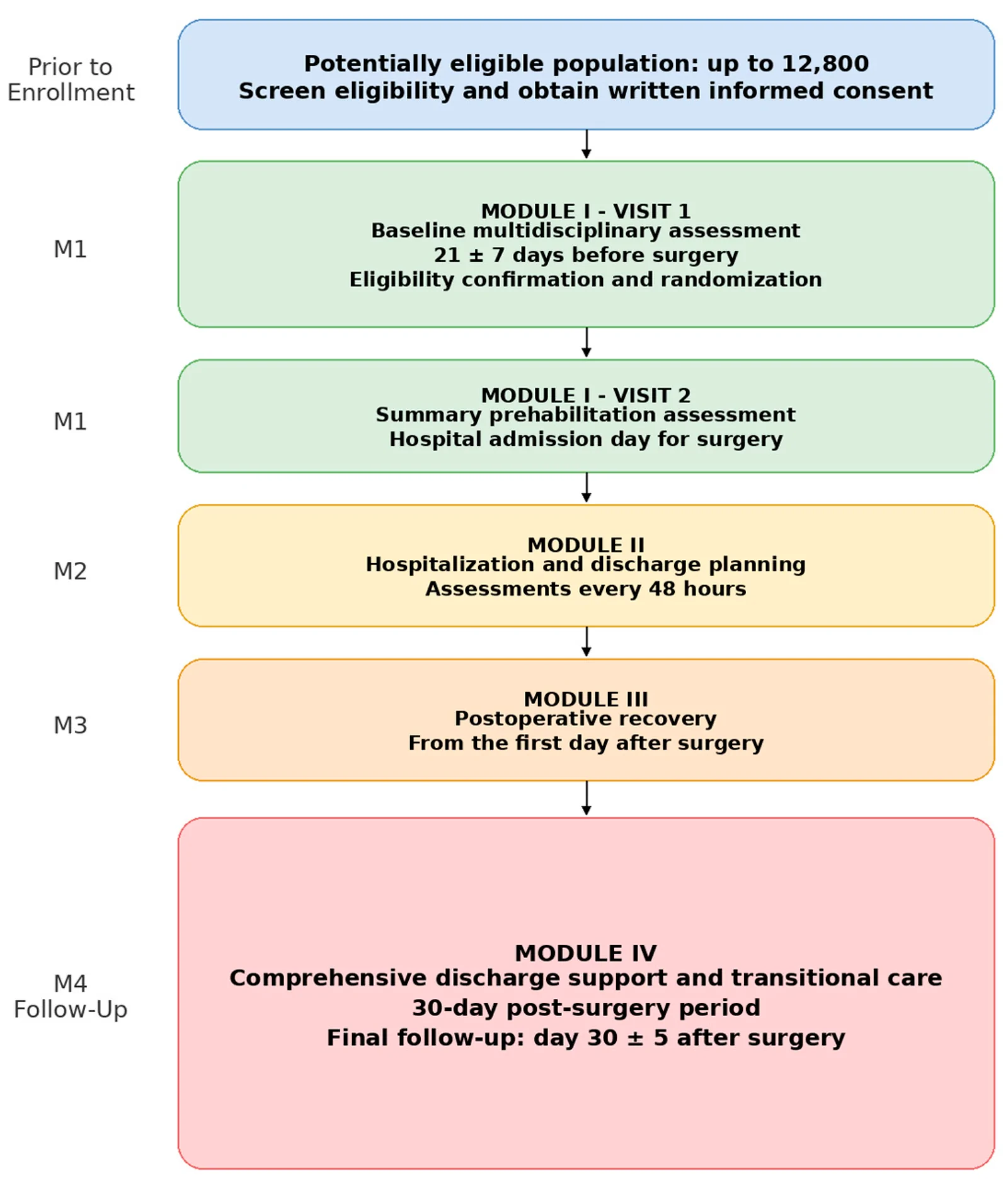
Flowchart of patient enrollment and participation.

The primary treatment effect of interest is the effect of assignment to the RESET pathway, compared with standard perioperative care, on each of the three primary outcomes: postoperative complications, prolonged index hospitalization, and 30-day unplanned hospital readmission. Each outcome will be evaluated separately within the oncology and non-oncology populations, yielding six primary hypothesis tests. Primary analyses will follow the intention-to-treat principle, with per-protocol analyses used as supportive assessments. The confirmatory decision rule and multiplicity control across all six outcome-by-population tests are specified in the Statistical Methods section of this paper. The overall study design and participant flow are presented in Figure 1.

### 2.2. Participants and Recruitment Procedure

#### Recruitment Scheme

Patients over 18 years of age who qualify for admission to surgical departments with an ICD-10 diagnosis requiring radical oncological surgery, as well as patients over 69 years of age referred for planned non-oncological surgical procedures, will be considered for inclusion. Eligible participants must meet all inclusion criteria and must not meet any of the exclusion criteria outlined below to be enrolled in the study.

## 3. Oncology Group

### 3.1. Inclusion Criteria

- Age ≥ 18 years;
- Eligibility for admission to a surgical department for radical cancer surgery based on an ICD-10 diagnosis;
- Preliminary eligibility for planned oncological surgical procedures, not solely diagnostic procedures, performed under general anesthesia;
- Informed consent to participate in the study.

### 3.2. Exclusion Criteria

- Age < 18 years;
- Eligibility for admission to a surgical department for diagnostic procedures to identify cancer based on an ICD-10 diagnosis;
- Pregnancy or breastfeeding.

## 4. Non-Oncology Group

### 4.1. Inclusion Criteria

- Informed consent to participate in the study;
- Age ≥ 70 years;
- All consecutive patients referred for planned non-oncological surgical procedures, not solely diagnostic procedures, performed under general anesthesia

### 4.2. Exclusion Criteria

- Cancer diagnosis.

Up to 12,800 potentially eligible patients will be approached and screened. At the stage of offering participation, approximately 25–35% of eligible patients are expected to decline; a 30% non-participation rate is assumed for planning purposes. Individuals who decline will not undergo study procedures, although routinely available clinical outcomes may be used in prespecified exploratory comparisons where permitted. Approximately 8960 participants are therefore expected to provide consent and be randomized. Randomized participants are allocated to the RESET intervention (90%) or standard-care control arm (10%). Both randomized arms undergo the protocol-defined assessment schedule; only the intervention arm receives the coordinated RESET pathway.

#### 4.2.1. Randomization

After written informed consent and confirmation of eligibility, each participant is assigned a unique study identification number. Randomization is performed centrally through a secure web-based system using a computer-generated dynamic allocation procedure based on a biased-coin algorithm. The procedure monitors cumulative allocation within each predefined clinical population and adjusts assignment probabilities as needed to maintain the protocol-defined 9:1 intervention-to-control ratio. Allocation is generated only after participant enrollment and eligibility confirmation and cannot be predicted or modified by Recruiting Investigators.

The unequal 9:1 allocation was chosen a priori for ethical and implementation reasons, principally to allow the greatest possible proportion of seriously ill surgical patients—particularly those undergoing oncological surgery—to receive the low-risk, multimodal RESET pathway while retaining a randomized standard-care comparator. The lower statistical efficiency associated with the smaller control group was taken into account in the approved sample-size planning described below. Under the simplifying assumption of equal outcome variances and a fixed total sample size, a 9:1 allocation has approximately 36% of the statistical efficiency of a 1:1 allocation for a simple two-group contrast (approximately 2.78-fold higher variance and 1.67-fold higher standard error). The substantially larger total recruitment target and prespecified covariate-adjusted analyses improve precision but do not eliminate this intrinsic efficiency penalty.

Randomization is conducted separately within the oncology and non-oncology populations. Allocation data are kept separate from participant-identifying and outcome data. No fixed block sizes or additional stratification factors are used beyond the separation of the two predefined clinical populations.

#### 4.2.2. Blinding

Because RESET is an individualized multidisciplinary care pathway, complete blinding is not feasible. In accordance with the approved study design and trial registration, participants are informed about study participation but are not explicitly told whether they have been allocated to the individualized RESET pathway or to standard perioperative care. Both groups undergo the same protocol-defined assessments, questionnaires, and activity monitoring. Some participants may nevertheless infer their allocation from the nature or intensity of care received; participant masking therefore refers to non-disclosure of randomized allocation rather than assurance that allocation cannot be recognized. Personnel delivering RESET are necessarily aware of allocation. Surgeons and other inpatient clinicians are not routinely informed of study allocation for research purposes, although information required for safe clinical care is never withheld. Outcome assessors and data analysts remain unaware of allocation whenever feasible. Allocation data are kept separate from outcome data, and the three primary outcomes are objective clinical endpoints.

### 4.3. Study Intervention

Of the approximately 8960 participants expected to be randomized, about 90% (approximately 8070) will be allocated to the RESET intervention arm.

The intervention consists of implementing the full procedure of the RESET tool. The following subinterventions are included as elements of the RESET tool:

- Prehabilitation Coordination System: The individual prehabilitation schedule for each study participant will be continuously monitored and coordinated by the research team members. The participant can report any additional needs or request consultations at any time.
- Nutritional Intervention: Nutritional status will be evaluated using anthropometric measurements, bioelectrical impedance analysis, and a clinical dietitian interview. Caloric and protein requirements will be calculated, and individualized dietary, supplementation, probiotic, or immunonutrition recommendations will be provided when clinically indicated.
- Pharmacological Intervention: Medications recommended through multidisciplinary consultations will be introduced if required by the patient. Vitamin supplementation will be added on the basis of the patient’s needs. For selected patients, probiotic supplementation will be introduced to optimize the gut microbiota.
- Physical Activity Intervention: A physiotherapist will conduct a detailed interview and functional assessment to identify the participant’s usual daily activity patterns, baseline exercise habits, physical capacity, individual needs, clinical condition, and requirements related to the planned surgery [3]. On this basis, individualized and comprehensive physical activity and exercise recommendations will be prescribed to optimize preoperative preparation and support the earliest possible recovery of function after surgery. Functional assessment will include the six-minute walk test, handgrip strength, FEV1, and the sit-to-stand test. Activity levels and implementation of the recommendations will be monitored using the wristband, mobile application, and follow-up assessments.
- Psychological Intervention: The Hospital Anxiety and Depression Scale (HADS) is used to screen for anxiety, depressive symptoms, and clinically relevant emotional distress. In accordance with the approved protocol, participants with a combined total HADS score (HADS-T; anxiety plus depression subscales; range 0–42) ≥ 15 are referred to a trained psychologist for further assessment and individualized support. This threshold applies to the total 14-item score and is distinct from cut-offs used for the anxiety and depression subscales separately. A HADS-T threshold of 15 has been validated as a clinically useful screening threshold for psychosocial distress in oncology populations [34]. Participants with a HADS-T score < 15 who have a relevant psychiatric history may be referred to a social worker for additional psychosocial support. The HADS score is used to identify patients who may benefit from further assessment; it is not, by itself, a psychiatric diagnosis. Subsequent management is based on clinical assessment and the participant’s psychosocial context. The psychologist may also use the Mini-Mental State Examination (MMSE) when clinically indicated. To reduce the risk of overlooking marked symptoms concentrated in one domain, HADS-A and HADS-D scores are also reviewed separately as part of clinical interpretation. These subscale scores do not define additional protocol referral thresholds; however, together with relevant psychiatric history and clinical assessment, they provide supplementary safeguards so clinically concerning anxiety or depressive symptoms are not disregarded solely because HADS-T is <15.
- Compliance Assessment: Wristband, Patient Application: Progress Monitoring Application. Each participant will receive a wristband to track physical activity. The patient application will provide a personalized prehabilitation plan on the basis of data collected during the first visit. This will enhance patients’ sense of security and improve their adherence to the prehabilitation team’s recommendations. It consists of the following modules:
  ○ Treatment Guidance and Education Module: Provides patients with information about the treatment plan and educates them about the disease and how to live with it;
  ○ Physiotherapy Module: Shares information about the recommended physical activity or exercises tailored to the patient, allowing them to track daily progress, such as recording step counts;
  ○ Nutrition Module: Provides the patient with individualized dietary recommendations regarding nutrient intake and special nutritional supplements;
  ○ Communication Module: Enables communication with the prehabilitation clinic coordinator.

Additionally, the RESET tool includes the following:

- Stochastic Organizational Support Network Planning: This is a model that reduces patient waiting times for specialist visits and improves resource allocation by optimizing niche specialist service availability;
- The Enhanced Recovery Protocol: This protocol focuses on maintaining nutrition, promoting early mobilization, and effective pain management [35,36,37];
- The Transitional Care Program: This program provides continuous monitoring and support after discharge to prevent rehospitalization, ensure follow-up care, and manage potential risks before complications arise [38].

Moreover, the SAFER Patient Flow Bundle [39] will be implemented to shorten patient stays and enhance the efficiency and safety of patient flow. The SAFER bundle is a practical strategy designed to minimize delays for adult patients in inpatient units. This practical tool combines five core best practices. Applying all five elements simultaneously is crucial for maximizing the overall benefits. When applied consistently, the SAFER bundle shortens patient stays and enhances the efficiency and safety of patient flow. The SAFER Patient Flow Bundle consists of the following:

- S (Senior Review): Every patient should receive a review by a senior clinician before midday. This clinician must have the authority to make care management and discharge decisions.
- A (All Patients): Each patient should be given an expected discharge date and clinical criteria for discharge, assuming an optimal recovery path with no unnecessary delays.
- F (Flow): Patient flow should begin promptly, transferring patients from assessment units to inpatient wards as early as possible.
- E (Early Discharge): The goal is for 33% of patients to be discharged from inpatient wards before noon.
- R (Review): A structured, multidisciplinary team assessment is required for patients with prolonged stays (over 7 days), with a focus on a “home first” approach.

Each of these elements is integral to achieving smoother patient transitions and optimizing hospital resources.

The SAFER bundle is particularly effective when implemented alongside the “Red to Green” system, a visual management tool designed to identify periods of inefficiency within a patient’s care pathway. A “Red Day” is defined as a day during which the patient receives minimal or no acute care that actively contributes to their treatment, whereas a “Green Day” indicates that the patient has received meaningful acute care that advances recovery and brings them closer to discharge [40,41].

All of the aforementioned interventions will be evaluated for their effectiveness in the final configuration of the RESET tool.

The effectiveness of the RESET tool will be assessed in comparison with the current treatment protocol representing standard care within the healthcare system. In this study, the standard preoperative patient pathway serves as the randomized comparator (control group). RESET does not evaluate an investigational medicinal product or an investigational medical device. Under standard practice, qualification for surgery and scheduling of hospital admission typically occur during a patient’s initial hospital outpatient clinic visit, which is made following a referral to a specific surgical department. In accordance with the requirements of the National Health Fund of Poland, patients are not obligated to attend this appointment in person; instead, they may be added to the waiting list based on a referral submitted electronically, by mail, or through telephone registration.

Ideally, preparation for treatment should begin during the patient’s first outpatient consultation. However, possession of a hospital referral does not automatically grant access to such a consultation. A separate referral to the appropriate specialty clinic is required. During the outpatient visit, the hospital physician—operating within a standardized time window for each patient—obtains a comprehensive medical history, including comorbidities, reviews diagnostic tests and imaging, orders additional examinations as needed, and prepares a hospital admission qualification sheet or other relevant documentation. The physician also screens for addictions, evaluates nutritional status, and collaborates with the patient to establish a preliminary treatment plan. Smoking cessation is encouraged, and intensive nutritional support may be prescribed. In cases of malnutrition or high nutritional risk, recommendations may include intensive nutritional therapy or, in severe cases, referral for percutaneous endoscopic gastrostomy placement. All of these activities must be completed within the allotted 15–20 min appointment duration.

In principle, patients awaiting surgery may pursue necessary specialist consultations; however, this is often difficult due to organizational constraints within the healthcare system and extended waiting times for certain outpatient services. Although the standard approach aims to identify perioperative risks and support appropriate preparation by surgical and anesthesia teams, its effective implementation is frequently challenging.

### 4.4. Intervention Delivery and Adherence

The RESET intervention consists of four coordinated modules delivered according to standardized protocols throughout the perioperative pathway. The key principle of the RESET program is individualized multidisciplinary care. Each participant allocated to the intervention group receives a personalized set of recommendations developed independently by each member of the prehabilitation team (physician, dietitian, physiotherapist, psychologist, pharmacist, and nurse coordinator). Consequently, although the intervention follows a standardized framework, the exact content and intensity of individual recommendations may differ between participants depending on their clinical condition, functional status, nutritional risk, psychological needs, comorbidities, and planned surgical procedure. The timing, sequence, and overall structure of all intervention components are summarized in Table 1 (*Schedule of activities*).

The prehabilitation module begins after enrollment and ordinarily covers the approximately 21 ± 7 days before surgery specified in the protocol. Nutritional counseling, physical activity recommendations, psychological support, medication review, management of modifiable risk factors, and patient education are provided according to identified needs. Components may be reinforced or modified during the preoperative period when clinically indicated.

On the day of hospital admission, the multidisciplinary team reassesses the participant’s health status, current needs, adherence to previous recommendations, and barriers to implementation. The findings are used to review and, when needed, individualize the perioperative management plan before surgery.

Participants in the intervention arm use the dedicated RESET mobile application to receive individualized recommendations and general information, view the schedule of visits and planned activities, communicate with the multidisciplinary prehabilitation team, and request support. Wristband and application data support monitoring of physical activity and the implementation of recommendations. Protocol-defined assessments and activity monitoring are also performed in the control arm for research purposes and are not components of the comparator intervention.

During hospitalization, adherence to enhanced recovery principles and standardized perioperative care is monitored continuously by the multidisciplinary team. After discharge, transitional care includes scheduled follow-up contacts, reinforcement of discharge recommendations, and monitoring for complications and rehospitalization.

Adherence is documented prospectively using the RESET digital platform, activity-monitoring wristband, follow-up assessments, and routine study documentation. Indicators include attendance at scheduled consultations, implementation of physical activity, nutritional, and medication-related recommendations, use of the application, and completion of planned follow-up activities. These process measures will be analyzed together with clinical outcomes to assess intervention fidelity, feasibility, and implementation.

### 4.5. Study Scheme

#### 4.5.1. Module I

**Initial Visit: Prehabilitation—Patient Admission and Initial Assessment (21 +/− 7 days before surgery)**

The initial visit includes presenting the potential participant with verbal and written information about the proposed study, providing a copy of the “Information for Study Participants” document, and providing a copy of a written informed consent form for participation in the study. At this stage, the participant should be fully informed by the Investigator about the study’s nature and schedule and their rights and responsibilities and receive detailed information from the Investigator on all relevant aspects, including the potential risks and benefits of participation.

Because eligibility can be confirmed without additional diagnostic testing solely for research purposes, the initial eligibility assessment may be combined with Visit 1. Participants who need additional time to review the informed consent form and participant information document may complete consent at a subsequent visit before any study-specific procedure is performed.

Every participant will be informed of their right to withdraw from the study at any stage without the need to provide a reason. Before any study procedure is initiated, the process of obtaining informed consent from the participant must be fully completed.

**Visit 1: Following Completion of Informed Consent Process**

1. The participant’s completion of the informed consent procedure will be documented, and the randomization process (Principal Investigator, Investigator) will be completed.

2. The Investigator will confirm in writing that the participant meets the inclusion/exclusion criteria on the designated study form.

3. Demographic and medical history interviews: The participant will respond to questions according to the study questionnaire, covering topics such as lifestyle habits, family history of cancer, and level of independence.

4. The American Society of Anesthesiologists (ASA) classification will be used to evaluate surgical risk related to serious complications or mortality during or after anesthesia.

5. A quality-of-life assessment using the SF-36 questionnaire will be provided to the patient, enabling them to self-evaluate their health status.

6. Pain intensity will be determined via the Numeric Rating Scale (NRS).

7. Physical functioning will be assessed via the Zubrod–ECOG–WHO scale (Eastern Cooperative Oncology Group), which is commonly used to evaluate the general condition and quality of life of cancer patients and in geriatrics, psychiatry, and chronic diseases.

8. The Charlson Comorbidity Index (CCI) will be used to estimate the 10-year survival probability in patients with multiple comorbidities.

9. Venous thromboembolism risk will be evaluated using the Caprini scale.

10. An assessment using the G8 scale for geriatric screening will be performed.

11. For older adults, a health status assessment will be conducted using the Vulnerable Elders Survey (VES).

12. The participant’s need for specialist consultations will be assessed (coordinated under SOSNP).

13. Blood will be collected for laboratory testing (CBC, electrolytes, albumin, cholesterol, total protein, triglycerides, TSH, FT3, FT4, iron, vitamin D3, AST, ALT, and GGTP) and biobanking. At the 4th Military Clinical Hospital, the protocol-defined allostatic load panel will additionally be assessed.

14. A nutritional assessment, including anthropometric measurements (BMI, WHR), a nutritional status evaluation using bioelectrical impedance analysis (BIA), and a clinical dietitian interview will be conducted.

15. A psychological evaluation will be conducted (HADS, MMSE).

16. Physiotherapy assessment: Grip strength (dynamometer), a six-minute walk test (6MWT), a FEV-1 test, and a sit-to-stand test (using a wristband) will be conducted.

17. A participant activity assessment will be conducted using a smart wristband. A mobile app will be installed on the participant’s smartphone that will store and cycle data from the wristband, such as step count, sleep duration, and heart rate. Training will be provided on wristband usage, measurement techniques, app functionality, and technical support.

18. Clinical pharmacist assessment: A medication review will be conducted.

19. Addiction-related re-education will be conducted (if applicable).

The collected data will serve as a baseline for evaluating study endpoints (initial assessments).

**Visit 2: Summary Prehabilitation Visit (Hospital Admission Day For Surgery)**

1. Health Status Update: A targeted interview will be conducted to assess any changes in the participant’s general health status since the first visit. A visit form will be completed, and scales will be reassessed for comparison with Visit 1 results.

2. Specialist Consultation Needs: An assessment will be conducted to identify any specialist consultation needs of the participant.

3. Treatment Plan Discussion: The treatment plan, including the surgery date and discharge schedule, will be discussed with the patient and caregiver (if applicable).

4. Nutritional Assessment: A dietary evaluation, anthropometric measurements (BMI, WHR), and a nutritional status assessment will be conducted using bioelectrical impedance analysis (BIA) and a clinical dietitian interview.

5. Psychological Assessment: A psychological assessment will be performed using the HADS.

6. Physical Therapy Assessment: Physical functionality will be evaluated through grip strength testing (with a dynamometer), the 6MWT, an FEV-1 test, and a sit-to-stand test with a smart wristband.

7. Activity Level Evaluation: The participant’s activity level will be assessed through data analysis using the data from the dedicated mobile application.

8. Clinical Pharmacist Evaluation: An assessment by a clinical pharmacist will be conducted.

9. Substance Cessation Assessment: An evaluation related to substance cessation will be conducted.

10. Medical Laboratory Diagnostics: Blood tests will include a CBC and measurement of electrolytes, albumin, cholesterol, total protein, triglycerides, TSH, FT3, FT4, iron, vitamin D3, AST, ALT, and GGTP. At the 4th Military Clinical Hospital, the protocol-defined allostatic load panel will additionally be assessed.

11. Participant Satisfaction Survey: A satisfaction survey will be conducted to assess the participant’s experience with the prehabilitation program.

#### 4.5.2. Module II

**Hospitalization and Discharge (Hospital Admission Day, with follow-ups every 48 h)**

1. Structured assessment of patient and caregiver needs, along with patient and caregiver education, including communication with the patient and family to establish a treatment schedule and set an expected discharge date.

2. Review of the prehabilitation protocol from Visit 2.

3. Evaluation and identification of patient needs related to mobility, nutrition, pharmacotherapy, and psychological support.

4. Assessment of the support capabilities of family members or caregivers.

5. Communication with a family member or caregiver who will provide posthospital assistance.

6. Providing the patient and caregiver with an overview of the hospital stay schedule, postprocedure activities, and discharge plan.

7. The patient’s stay in the ward will adhere to the SAFER Patient Flow Bundle.

#### 4.5.3. Module III

**Postoperative Recovery (From the first day after the surgical procedure)**

1. Surgical Procedure and Hospitalization Assessment:
  - Health Assessment: Regular monitoring of health parameters and treatment progress;
  - Registration of the ICD-10 and ICD-9 procedure codes;
  - Clavien–Dindo classification for complications;
  - Evaluation of perioperative pharmacotherapy;
  - Registration of anesthesia type;
  - Monitoring of postanesthesia complications;
  - Wound healing assessment via the VAS scale.
2. Enhanced Recovery Protocol (early patient mobilization, bedside rehabilitation, gradual physical activation, nonstandard dietary management, additional rehabilitation support).
3. Daily Red to Green assessment.
4. Evaluation of family communication needs.
5. The patient’s stay in the ward will adhere to the SAFER Patient Flow Bundle.

#### 4.5.4. Module IV

**Comprehensive Discharge Support System (30-day Post-Surgery Period)**

1. Transitional Care Program:
  - Contact with primary care providers and community nurses;
  - Scheduling of oncological consilium;
  - Development of discharge and long-term care recommendations.
2. Reinforcement of SAFER Patient Flow Bundle standards, with coordination between the social worker and a nurse to establish optimal conditions for early discharge and ensure an individualized transitional care program.
3. Monitoring and Analysis of Prolonged Hospitalizations and Rehospitalizations:
  - Analysis of the reasons for PH/RH;
  - Review of treatments administered;
  - Assessment of therapeutic outcomes achieved;
  - Evaluation of the impact on the patient’s overall condition.

**Follow-up Visit (Day 30 ± 5 after surgery)**

A follow-up interview will be conducted at day 30 ± 5 after surgery to assess changes in general health status compared with Visit 1, document the postoperative course, and repeat applicable scales. The follow-up may be conducted by telephone or on site at the study center.

- For an on-site follow-up visit, the histopathology results and treatment outcomes will be reviewed when applicable.
- Nutritional assessment will include anthropometric measurements (BMI and WHR), bioelectrical impedance analysis, and consultation with a clinical dietitian.
- Psychological assessment will be conducted using the HADS.
- Physical-function assessment will include handgrip strength, the six-minute walk test, FEV1, and the sit-to-stand test.
- A clinical pharmacist assessment will be performed.

The examination of participants will take place in accordance with the table below (Table 1).

## 5. Outcome Measures

The three primary outcomes are objectively defined clinical measures: prolonged index hospitalization, unplanned hospital readmission, and postoperative complications. Prolonged index hospitalization is defined as an index hospital stay lasting ≥ 14 calendar days from admission to discharge. Readmission is defined as an unplanned admission to the same or another hospital within 30 days after discharge from the index hospitalization. Postoperative complications are assessed for 30 days after the index surgical procedure.

### 5.1. Primary Endpoints

1. Participants with prolonged index hospitalization.

The outcome will be reported as the number and percentage of participants whose index hospitalization lasts at least 14 calendar days. The number of days beyond day 14 and the number of consecutive clinically unjustified Red days at the end of hospitalization will also be recorded.

2. Participants with an unplanned hospital readmission within 30 days after index hospital discharge.

Readmissions will be identified using electronic health records, hospital information systems, and participant follow-up interviews. The outcome will be reported as the number and percentage of participants with at least one unplanned readmission within 30 days after discharge.

Prolonged hospitalization and readmission will be compared between the RESET and standard-care arms. Additional analyses will describe the duration and reasons for prolonged hospitalization, time to readmission, causes of readmission, associated treatment, impact on the participant’s condition, and related costs.

3. Participants with at least one postoperative complication within 30 days after surgery.

Postoperative complications will be identified and graded using the Clavien–Dindo classification. The outcome will be reported as the number and percentage of participants with at least one Clavien–Dindo grade I-V complication within 30 days after surgery. For participants with multiple complications, the highest grade observed during this period will additionally be recorded.

### 5.2. Secondary Endpoints

1. Length of hospital stay.

Length of index hospital stay will be measured in calendar days from admission for the index surgical hospitalization to discharge. In accordance with the approved protocol, a prespecified secondary analysis will compare length of stay between randomized groups among “cases without complications.” The protocol does not further operationalize this phrase. The statistical analysis plan (SAP) will prospectively define this subgroup, before database lock and without examination of treatment effects, as participants who remain free of a Clavien–Dindo postoperative complication during the index hospitalization before discharge. This definition is intentionally distinct from the primary complication outcome, which is assessed through postoperative day 30. Because subgroup membership is determined after randomization and may itself be affected by RESET, restriction to this subgroup does not preserve the randomized comparison and may introduce selection bias; a causal interpretation of the between-group difference is therefore not available. The analysis will be reported as a conditional, descriptive/associational comparison.

The overall effect of randomized assignment on index-hospital length of stay will be assessed separately in a complementary intention-to-treat analysis including all randomized participants, irrespective of postoperative complications. If the distribution of length of stay does not meet the assumptions of conventional linear models, regression methods appropriate to its distribution will be used.

2. Overall health status of patients.

Overall health status will be evaluated as a multidomain secondary outcome using protocol-defined measures: patient-reported and clinical scales, including SF-36 and HADS; laboratory parameters and allostatic load measures; physical-function tests, including the six-minute walk test, handgrip strength, FEV1, and sit-to-stand performance; and anthropometric and nutritional measures, including BMI, WHR, and bioelectrical impedance analysis. Each component will be analyzed separately using its validated score or original measurement unit. Changes from baseline (21 ± 7 days before surgery) to hospital admission and to follow-up at day 30 ± 5 after surgery will be compared between study arms, where the applicable assessment is completed. HADS anxiety and depression subscale scores will additionally be reported separately using their validated scoring conventions, while the protocol-defined HADS-T ≥ 15 threshold remains the clinical referral criterion.

3. Patient satisfaction.

Patient satisfaction will be assessed using a dedicated questionnaire. Patient satisfaction affects adherence to medical recommendations and overall perceptions of healthcare system effectiveness.

4. Compliance.

Compliance will be monitored via wristbands, the mobile application, and follow-up visits. Adherence to prehabilitation recommendations is clinically relevant to the evaluation of the RESET pathway and will therefore be documented prospectively as a secondary and implementation outcome.

5. Effectiveness of Stochastic Organizational Support Network Planning.

The number of interventions within the SOSNP and participants’ waiting times for the aforementioned specialist consultations will be compared to the average patient waiting time in the standard NHF system.

6. Costs associated with RESET.

Treatment costs will be measured on the basis of the analysis of costs associated with hospitalization, prehabilitation, rehabilitation, postoperative follow-up visits, and any PH/RH, as well as financial effectiveness indicators. A comparative analysis of treatment costs between patients in the RESET intervention group and those in the control group will also be conducted.

### 5.3. Study Conditions and Analytic Strategy

The study includes two clinical populations, oncology and non-oncology, each randomized to intervention or control. Prespecified exploratory analyses may additionally include a naturally formed non-participant comparison group for routinely available clinical outcomes. Repeated outcomes measured at two or more protocol-defined time points will be analyzed using models that account for within-participant change, between-group effects, and relevant covariates. Outcomes measured once will be analyzed using endpoint-appropriate regression models.

The analysis has two complementary tracks. The primary randomized track comprises randomized comparisons of the RESET and standard-care arms. A secondary exploratory track may compare routinely available outcomes—prolonged hospitalization, readmission, length of stay, and postoperative complications—with the non-participant group. Patient-reported and participant-specific outcomes, including multidomain health status, satisfaction, and adherence, will be analyzed only among consenting randomized participants.

Exploratory comparisons with eligible non-participants will be kept separate from the randomized analyses. Because RESET includes clinically heterogeneous operations and specialty-specific risk profiles, a single propensity-score model would not be appropriate across all surgical populations. Where propensity-score methods are used, the model will be defined prospectively within clinically coherent groups before outcomes are compared. Adjustment will be limited to characteristics recorded before the decision to participate and available in a comparable form for both participants and non-participants. The covariate set will be selected from routinely available demographic, center, calendar-period, and procedural characteristics, with additional specialty- or procedure-specific baseline factors included when clinically relevant and consistently available. Variables arising after study participation will not be used. Covariate balance and overlap between groups will be checked before adjusted comparisons are interpreted. These analyses will remain exploratory and will not influence the primary randomized treatment-effect analyses. For transparency, the prespecified core covariate set for any propensity-score analysis will comprise age, sex, study site, calendar year, ASA physical status class, Charlson Comorbidity Index, and broad surgical-procedure category. Propensity-score analyses will be undertaken only within clinically coherent groups in which these core variables are recorded before the decision to participate and are available in a comparable form for participants and eligible non-participants. Any additional specialty- or procedure-specific baseline covariate must be clinically justified and defined before outcome comparison and must be consistently available in both groups.

Analyses of accumulated data may be conducted annually to support implementation monitoring and prespecified interim publications. Final analyses will be performed after completion of follow-up. Study year and site may be included as covariates to evaluate temporal and center-related variation; these analyses will not replace the prespecified randomized comparison.

### 5.4. Sample Size

The approved protocol specifies a two-sided significance level of α = 0.01 and a target statistical power of 95% for the primary analyses. The recruitment target itself, however, was constrained primarily by feasible surgical volumes rather than obtained by solving a single power equation. Based on 2023 surgical volumes and anticipated pre-randomization non-participation, approximately 5740 oncological participants (about 570 control and 5170 RESET) and 3220 non-oncological participants aged ≥70 years (about 320 control and 2900 RESET) are expected to be randomized, for an overall randomized sample of approximately 8960.

The approved protocol also reported reference calculations for selected statistical models. For logistic regression of prolonged hospitalization/readmission, an odds-ratio magnitude of 1.30, Pr(Y = 1 | X = 1) under H0 = 0.20, two-sided α = 0.01, and 95% power yielded *N* = 1632 in G*Power version 3.1.9.7 [42]. A repeated-measures MANOVA reference calculation for multidomain health-status outcomes (Pillai’s V = 0.01) yielded *N* = 1721. The protocol did not contain three independently derived endpoint-specific calculations, nor a separate original event-rate assumption or power calculation for postoperative complications.

To provide the endpoint-specific justification requested by the Reviewer and to characterize the fixed design transparently, Table 2 additionally reports current prospective operating-characteristic benchmarks for each primary outcome and clinical population. These calculations do not alter the approved recruitment target or reconstruct the original protocol calculation. They use the planned 9:1 group sizes, 95% power, and the most stringent first-step Holm threshold for the six-test family (two-sided α = 0.01/6 = 0.00167). Plausible control-event-rate scenarios were selected by the Investigators from participating-center historical experience and the literature considered during trial planning. The calculations use the normal approximation for two independent proportions without continuity correction.

Because Holm’s procedure is step-down, α = 0.00167 is a conservative single-hypothesis benchmark rather than the threshold applied to every ordered test. The detectable effects in Table 2 should therefore be interpreted as worst-step design-sensitivity benchmarks; the full family-wise operating characteristics depend on the joint pattern of the six primary *p* values. The SAP, finalized before database lock and before treatment effects are examined, will document these assumptions and may supplement them with simulation-based scenarios using blinded pooled event-frequency information. This prospective characterization will not alter the recruitment target, allocation ratio, endpoints, or protocol-defined α and power targets.

The protocol did not provide a detailed narrative rationale for the α = 0.01 and 95% power combination, so the original specification is distinguished here from the explanatory rationale added in this revision. Retaining a stringent α reduces the risk that isolated primary findings are over-interpreted in a trial with multiple primary comparisons and an extensive secondary and implementation program, while the 95% power target reflects a preference for stronger assurance that clinically meaningful effects are not missed. With the fixed, large recruitment base, this conservatism translates into a larger minimum detectable effect rather than a larger recruitment target: for prolonged hospitalization in the oncology population, the detectable absolute reduction is approximately 4.6 percentage points under α = 0.05 with 80% power, 6.8 percentage points under α = 0.01 with 95% power, and 7.6 percentage points using the conservative first Holm step at α = 0.00167 with 95% power. The Investigators considered this evidential standard appropriate because RESET is intended to inform system-level implementation and potentially reimbursement, where false-positive conclusions would have substantial organizational and financial consequences.

### 5.5. Data Collection and Validation

Data will be collected by trained personnel using standardized templates designed to capture all required information and ensure accurate patient identification. The Principal Investigator will supervise data cataloging and oversee adherence to data management procedures. The templates will be based on a methodological catalog that has been in continuous use at the hospital for approximately 20 years and is regularly updated to maintain accuracy and relevance. All metadata will be stored in a secure system for future use, verification, or publication, with access restricted to authorized personnel only.

The research dataset will comprise multiple data types—including narrative descriptions, numerical calculations, graphic files, and outputs from medical devices—and is expected to total no more than 0.5 TB. To ensure high data quality, the study will implement a comprehensive set of quality control procedures. These include real-time validation at the point of data entry (e.g., automated field checks), logical and consistency analyses, continuous monitoring and auditing to identify deviations from established standards, proactive management of missing data through timely detection and corrective measures, and statistical procedures to identify outliers and evaluate trends. Personnel responsible for data collection and entry will undergo dedicated training, and all procedures will be validated and pilot-tested on small samples prior to full-scale implementation. Collectively, these measures will ensure that the resulting dataset is accurate, reliable, and suitable for subsequent analyses and applications.

### 5.6. Statistical Methods

A detailed statistical analysis plan will be finalized before database lock and before the final between-group analyses. It will retain the protocol-defined populations, 9:1 randomization ratio, primary and secondary outcomes, sample-size assumptions, and significance threshold, while setting out the planned adjusted, supportive, sensitivity, and missing-data analyses in greater detail.

The oncology and non-oncology populations are predefined and will be analyzed separately. For each primary outcome, the principal analysis will estimate the effect of assignment to the RESET pathway compared with standard perioperative care during the protocol-defined follow-up period.

The three primary outcomes are separate primary outcomes rather than co-primary endpoints requiring simultaneous significance. Each outcome will be evaluated separately within each of the two predefined clinical populations, yielding six outcome-by-population primary hypothesis tests. No hierarchical or gatekeeping order and no composite primary endpoint are imposed. The multiplicity-controlled decision rule for confirmatory conclusions is specified below.

Primary randomized analyses will follow the intention-to-treat principle. All randomized participants will be analyzed in the group to which they were assigned, regardless of incomplete uptake of RESET, discontinuation or limited use of the mobile application or activity-monitoring wristband, missed prehabilitation consultations, or incomplete adherence to individual recommendations. These events will be recorded as measures of adherence and implementation but will not lead to reassignment or exclusion from the intention-to-treat analysis.

Per-protocol analyses will be performed as supportive analyses. The criteria for the per-protocol population will be defined prospectively in the statistical analysis plan before treatment effects are examined and will be based on major eligibility, randomization, or intervention-delivery deviations. Adherence will also be evaluated as a protocol-defined secondary and implementation outcome.

Each of the three binary primary outcomes—prolonged index hospitalization, unplanned hospital readmission within 30 days after discharge, and at least one postoperative complication within 30 days after surgery—will be analyzed using logistic regression. Numbers and proportions of events will be reported for each randomized group together with treatment-effect estimates and confidence intervals.

Adjusted analyses will use a parsimonious set of baseline factors with clear prognostic relevance—age, sex, study site, study year, ASA class, Charlson Comorbidity Index, and broad surgical-procedure category—subject to final prospective specification in the statistical analysis plan. Unadjusted randomized comparisons will also be reported. Measures of adherence or intervention exposure recorded after randomization will not be used to adjust the principal treatment-effect models.

Repeated outcomes will be analyzed primarily using mixed-effects models appropriate to the measurement scale. Continuous repeated outcomes will be assessed with linear mixed-effects models, and binary, ordinal, or count repeated outcomes with generalized linear mixed-effects models using an appropriate link function. Models will include randomized group, assessment time point, and the group-by-time interaction, with a participant-level random intercept to account for within-participant correlation. Baseline handling, the covariance structure, any random slopes, the treatment of study site, and any additional prespecified prognostic covariates will be defined in the SAP before treatment effects are examined. Repeated-measures ANCOVA will be restricted to supportive or sensitivity analyses.

Effect estimates will be reported with confidence intervals in addition to *p* values. The protocol-defined two-sided α = 0.01 threshold will be retained as the nominal design threshold, and principal primary-effect estimates will be accompanied by 99% confidence intervals. Consistent with the protocol, nominal *p* values between 0.01 and 0.05 may be described as exploratory. For the six primary hypotheses, however, confirmatory status will be determined by the multiplicity-adjusted decision rule below; any primary result that does not satisfy that rule will be considered non-confirmatory irrespective of its nominal *p* value.

The approved protocol specified α = 0.01 but did not define a formal family-wise multiplicity procedure. Because each of the three primary outcomes will be tested separately in each of the two predefined clinical populations, the confirmatory primary family comprises six outcome-by-population hypothesis tests. As a prospective safeguard specified before database lock and before examination of treatment effects, Holm’s step-down procedure will be applied across all six primary *p* values to control the family-wise error rate at α = 0.01 across both outcomes and clinical populations. A confirmatory positive finding for a specific outcome-population hypothesis will require an estimated effect in the prespecified direction favoring RESET and a Holm-adjusted two-sided *p* value < 0.01. Simultaneous significance of all three outcomes is not required. Rejection of one or more hypotheses under this procedure will support a confirmatory conclusion only for the corresponding endpoint(s) and clinical population(s); it will not imply benefit for the remaining primary outcomes or for the other population. All six primary results will be reported regardless of statistical significance.

The amount and reasons for missing data will be summarized by randomized group and clinical population. Primary outcomes will be obtained from the protocol-defined sources, including electronic health records, hospital information systems, and scheduled follow-up, subject to applicable consent and data-protection requirements. The approach to missing data will depend on their extent, pattern, and likely mechanism. Longitudinal models will use all available observations under their stated assumptions. Multiple imputation will be considered when appropriate, particularly when a missing-at-random assumption is clinically and statistically plausible and suitable auxiliary information is available. If missing primary-outcome data are substantial, sensitivity analyses will examine whether the conclusions remain robust under alternative assumptions. Complete-case analyses may be reported as supportive analyses but will not replace the intention-to-treat analysis.

Analyses performed while recruitment and follow-up are ongoing, whether for implementation monitoring or interim scientific reporting, will be descriptive or exploratory and will not be used for formal efficacy stopping decisions. The final protocol-defined primary analyses will be performed after completion of the planned follow-up and database lock.

Data management and preprocessing will be conducted using spreadsheet software and R (version 4.4.2 or higher) [43], with established R packages including the tidyverse ecosystem [44]; JASP or Jamovi may be used as complementary tools.

During revision in response to peer review, ChatGPT (OpenAI, GPT-5.6 Sol) was used to assist with language editing and drafting of revised methodological descriptions. It was not used to generate or analyze study data. All methodological and statistical decisions and the final wording were reviewed and approved by the authors, who take full responsibility for the manuscript.

### 5.7. Protocol Compliance

A protocol deviation refers to any instance where the study protocol is not followed, such as administering an incorrect intervention or misapplying eligibility criteria. Significant deviations are characterized by cases where participants fail to provide informed consent or intentionally submit inaccurate information. Additional examples of protocol deviations include errors in participant randomization or instances where individuals in the intervention group do not receive the specified RESET program components.

### 5.8. Participant Confidentiality

The Principal Investigator is responsible for ensuring participant confidentiality. All participant records and information will be pseudo-anonymized using unique identification codes to maintain privacy. Electronic or physical data that could identify participants will be securely stored either on a restricted-access server, accessible only to authorized study personnel, or in locked filing cabinets. Anonymized data will be retained for 20 years and subsequently disposed of securely as confidential waste.

### 5.9. Ethical Approval

The study is conducted in accordance with the Declaration of Helsinki, applicable Polish regulations, Good Clinical Practice principles, and the approved protocol. Ethical approval was granted by the Bioethics Committee at the Lower Silesian Medical Chamber in Wroclaw (approval No. 33/BNBO/2025, 12 November 2025). Important protocol modifications will be submitted to the Bioethics Committee, incorporated into study documentation, and reflected in the public registry and subsequent publications when applicable.

In addition to the randomized cohort, the approved study framework permits exploratory analyses of routinely collected clinical outcomes among otherwise eligible patients who decline participation. These patients are not enrolled in RESET, undergo no study-specific procedures, and receive no study-specific intervention. The non-participant component is therefore a secondary record-based analysis of existing clinical data. Under Chapter 4 of the Polish Act of 5 December 1996 on the Professions of Physician and Dentist (consolidated text: Journal of Laws of 2026, item 37, as amended) [45], Article 21 defines medical experiments conducted on humans and Article 29 requires a positive opinion on a medical-experiment project from an independent bioethics committee. Because this component involves no study-specific intervention, patient contact, or collection or analysis of biological material for research, it does not meet the statutory definition of a medical experiment. Accordingly, a separate positive bioethics-committee opinion under Article 29 and study-specific research consent are not required under the cited provisions. The analysis will be conducted in accordance with the Declaration of Helsinki and applicable data-protection requirements. These non-participant analyses will remain exploratory and will not contribute to the primary randomized treatment-effect estimates.

### 5.10. Informed Consent

Written informed consent is obtained from every participant before enrollment and before any study-specific procedure is performed. Participants may withdraw consent at any time without providing a reason and without affecting their clinical care.

**Trial registration**

The RESET study is registered at ClinicalTrials.gov (NCT07727876; Unique Protocol ID 2024/ABM/02/00051). Recruitment began on 16 February 2026. The registry record was first submitted on 17 July 2026 and first posted on 27 July 2026; registration therefore occurred after recruitment had commenced.

The study is a medical research experiment under the applicable Polish framework and does not evaluate an investigational medicinal product or an investigational medical device; therefore, it is not registered in the European Union Clinical Trials Information System (CTIS).

## 6. Discussion

RESET evaluates an integrated perioperative pathway combining individualized prehabilitation, coordinated inpatient care, structured discharge planning, and transitional care. Existing evidence suggests that prehabilitation can improve functional capacity and selected postoperative outcomes, but effects vary across populations, intervention content, and implementation settings [1,4,6,7,8,9,10,11,12,13,14,15,16]. The present trial therefore tests the effectiveness of the integrated pathway rather than assuming that benefits of individual elements will translate directly to the complete RESET model.

The clinical rationale is particularly relevant to patients with cancer and older surgical patients, in whom limited physiological reserve, frailty, multimorbidity, nutritional deficits, and short preoperative windows may modify response to optimization. Prolonged hospitalization and readmission are also multifactorial outcomes that reflect perioperative morbidity, discharge preparation, access to follow-up, patient and caregiver support, and organizational factors [29,30,31]. RESET addresses these domains within one coordinated pathway spanning the preoperative, inpatient, and 30-day post-discharge periods.

The 9:1 allocation increases experience with delivery and implementation of RESET while preserving a randomized standard-care comparator, but the smaller control group necessarily reduces statistical efficiency compared with balanced allocation. The protocol’s generic reference calculations should therefore not be interpreted as guaranteeing 95% power for every endpoint-population hypothesis. The current manuscript reports conservative endpoint-specific design-sensitivity benchmarks under the planned allocation and the first Holm step; the SAP will document the full family-wise operating characteristics before database lock and before treatment effects are examined.

Structured discharge and transitional-care models, including patient-flow and post-discharge support strategies, provide evidence that organizational interventions can reduce avoidable delay and readmission in selected settings [46,47,48,49,50,51,52,53,54]. RESET extends these concepts by integrating them with prehabilitation and enhanced-recovery principles. Economic and organizational outcomes are included because prolonged hospitalization, complications, and readmission consume hospital capacity and increase costs; these analyses will evaluate, rather than assume, whether the integrated pathway provides system-level benefit. The system-level relevance also extends beyond direct hospital expenditure. Polish estimates indicate that cancer-related sickness-absence costs increased from PLN 2.6 billion in 2015 to PLN 4.6 billion in 2022, while projected productivity losses associated with cancer mortality exceed PLN 13 billion annually [55].

The trial estimates the effect of the bundled RESET pathway rather than the causal effect of individual components. Process measures such as adherence, intervention fidelity, specialist access, and use of digital tools may characterize how the pathway is delivered, but they cannot identify which component is causally responsible for any observed treatment effect.

Several limitations should be considered. Intervention providers cannot be blinded, some participants may infer allocation, surgical procedures are heterogeneous, and exploratory comparisons with non-participants remain susceptible to residual selection bias. In addition, the protocol-defined length-of-stay analysis among cases without complications defines a subgroup after randomization. The SAP will prospectively operationalize this subgroup as participants remaining free of a Clavien–Dindo postoperative complication during the index hospitalization before discharge. Because this status may itself be affected by the intervention, restriction to the subgroup may introduce selection bias and does not preserve the randomized comparison; a causal interpretation of the conditional between-group difference is therefore not available. The analysis will be interpreted descriptively/associationally, whereas a complementary all-randomized intention-to-treat analysis will provide the overall treatment-effect estimate for index-hospital length of stay. These limitations will be addressed through prospective adjusted and sensitivity analyses, blinded outcome assessment where feasible, and transparent separation of randomized and exploratory analyses.

## 7. Trial Status

At the time of this revision, the RESET trial remains ongoing and is actively recruiting participants.

Protocol version: 1.2, 27 March 2026

Recruitment start: 16 February 2026 (actual)

Primary completion: 16 May 2030 (anticipated); study completion: 31 July 2031 (anticipated)

## Figures and Tables

**Table 1:** Schedule of activities.

Procedure/Activity	Module I—Visit 1 21 ± 7 Days Before Surgery	Module I—Visit 2 Hospital Admission	Module II Hospitalization and Discharge	Module III Postoperative Recovery	Module IV 30-Day Post-Surgery Period	Final Visit 30-Day Post-Surgery Follow-Up
Informed consent	**X**					
Inclusion/Exclusion Form	**X**					
Demographics	**X**					
Medical history	**X**					**X**
Randomization	**X**					
RESET intervention	**X**	**X**	**X**	**X**	**X**	
Physical exam	**X**	**X**	**X**	**X**	**X**	**X**
Medical Laboratory Diagnostics	**X**	**X**	**X**	**X**		**X**
Nutritional assessment	**X**	**X**				**X**
Psychological evaluation	**X**	**X**				**X**
Physiotherapy assessment	**X**	**X**				**X**
Clinical pharmacist assessment	**X**	**X**				**X**
Addiction-related re-education (if applicable)	**X**					
Installation of a mobile app + wristband	**X**					
Activity Level Evaluation		**X**				**X**
Medical Scales Assessment	**X**	**X**	**X**	**X**	**X**	**X**
Treatment plan discussion		**X**				
Participant Satisfaction Survey		**X**				**X**
SAFER Bundle		**X**	**X**	**X**	**X**	
Red to Green		**X**	**X**	**X**	**X**	
Early Mobilization Program				**X**		
Transitional Care Program					**X**	
Complete Visit Forms	**X**	**X**	**X**	**X**	**X**	**X**

**X**, scheduled protocol-defined activity or assessment.

**Table 2:** Conservative endpoint-specific design-sensitivity benchmarks for the fixed 9:1 randomized design.

Population	Primary Outcome	Assumed Control Risk	Detectable RESET Risk	Absolute Reduction	Odds Ratio	Relative Reduction
Oncology	Prolonged index hospitalization	20.0%	12.4%	7.6 pp	0.57	38%
Oncology	30-day readmission	12.0%	6.2%	5.8 pp	0.49	48%
Oncology	Postoperative complications	30.0%	21.0%	9.0 pp	0.62	30%
Non-oncology	Prolonged index hospitalization	15.0%	6.7%	8.3 pp	0.41	55%
Non-oncology	30-day readmission	10.0%	3.5%	6.5 pp	0.33	65%
Non-oncology	Postoperative complications	25.0%	14.2%	10.8 pp	0.50	43%

Note: Oncology calculations use approximately *n* = 570 control and *n* = 5170 RESET; non-oncology calculations use approximately *n* = 320 control and *n* = 2900 RESET. Benchmarks assume 95% power and a two-sided α = 0.00167, corresponding to the most stringent first step of Holm’s procedure across six primary tests. Assumed control risks are planning scenarios, not observed trial event rates.

## Data Availability

De-identified individual participant data underlying published results, together with the associated data dictionary, study protocol, and statistical code where applicable, may be made available to qualified researchers upon reasonable request after publication of the primary results. Access will be subject to participant consent, applicable data-protection requirements, Sponsor and institutional approval, a methodologically sound proposal, and execution of a data-use agreement. Authorized representatives from the Sponsor, host institution, and regulatory authorities will retain direct access to study-related records for monitoring, auditing, and inspection purposes as required.

## Abbreviations

6MWT	Six-Minute Walk Test
ALT	Alanine aminotransferase
ASA	American Society of Anesthesiologists
AST	Aspartate aminotransferase
BIA	Bioelectrical Impedance Analysis
BMI	Body Mass Index
CBC	Complete Blood Count
CCI	Charlson Comorbidity Index
FEV-1	Forced Expiratory Volume in 1 Second
FT3	Triiodothyronine
FT4	Thyroxine
G8	Scale for geriatric screening
GDP	Gross domestic product
GGTP	Gamma-glutamyl transpeptidase
HADS	Hospital Anxiety and Depression Scale
HDL	High-Density Lipoprotein
ICD-10	10th revision of the International Classification of Diseases
ICH GCP	International Council for Harmonisation Good Clinical Practice
LDL	Low-Density Lipoprotein
MMSE	Mini-Mental State Examination
NIS	National Inpatient Sample
NRS	Numeric Rating Scale
PH	Prolonged hospitalization
RH	Rehospitalization (readmission)
SAFER	Safe Admission Flow and Efficient Routing Protocol
SF-36	36-Item Short Form Survey
SOSNP	Stochastic Organizational Support Network Planning
TSH	Thyrotropin
VES	Vulnerable Elders Survey
WHR	Waist to Hip Ratio
